# *Trichodesmium* – a widespread marine cyanobacterium with unusual nitrogen fixation properties

**DOI:** 10.1111/j.1574-6976.2012.00352.x

**Published:** 2012-09-20

**Authors:** Birgitta Bergman, Gustaf Sandh, Senjie Lin, John Larsson, Edward J Carpenter

**Affiliations:** 1 Department of Botany, Stockholm UniversityStockholm, Sweden; 2 Department of Marine Sciences, University of ConnecticutGroton, CT, USA; 3 Romberg Tiburon Center for Environmental Studies, San Francisco State UniversityTiburon, CA, USA

**Keywords:** cyanobacteria, N_2_ fixation, genome evolution, diazocytes, adaptation, nutrient stress

## Abstract

The last several decades have witnessed dramatic advances in unfolding the diversity and commonality of oceanic diazotrophs and their N_2_-fixing potential. More recently, substantial progress in diazotrophic cell biology has provided a wealth of information on processes and mechanisms involved. The substantial contribution by the diazotrophic cyanobacterial genus *Trichodesmium* to the nitrogen influx of the global marine ecosystem is by now undisputable and of paramount ecological importance, while the underlying cellular and molecular regulatory physiology has only recently started to unfold. Here, we explore and summarize current knowledge, related to the optimization of its diazotrophic capacity, from genomics to ecophysiological processes, via, for example, cellular differentiation (diazocytes) and temporal regulations, and suggest cellular research avenues that now ought to be explored.

## Introduction

Balancing of nitrogen (N) inputs and exports in global oceans requires substantial biogenic fixation of atmospheric nitrogen (N_2_). In this context, planktonic colony-forming cyanobacteria of the genus *Trichodesmium* are recognized as major players. Representatives within the genus have consistently been shown to be stable components of tropical and subtropical segments of the Atlantic, Pacific, and Indian Oceans where they may form enormous surface accumulations (‘blooms’) visible to the naked eye (see Capone & Carpenter, 1982; Capone *et al*., 1998; Karl *et al*., 2002; Tyrrell *et al*., 2003; Davis & McGillicuddy, 2006; Westberry & Siegel, 2006; Carpenter & Capone, 2008). *Trichodesmium* contributes to sustaining marine life via active release of key nutrients, for example carbon and nitrogen, and upon death and decay, hence making this fully photoautotrophic genus a vital player in the biogeochemical cycling of basic elements in contemporary oceans (Carpenter & Capone, 2008). The global input via N_2_ fixation by *Trichodesmium* was initially estimated to amount to about 5 Tg N annually by Capone & Carpenter (1982), an estimate that by now has risen to about 60–80 Tg N annually (Capone *et al*., 1997; Mahaffey *et al*. 2005; Westberry & Siegel, 2006; Carpenter & Capone, 2008), which makes up a substantial part of the current estimate of global marine N_2_ fixation, 100–200 Tg N annually (Karl *et al*., 2002).

As the N_2_-fixing enzyme, nitrogenase, encoded by the *nifHDK* genes, is rapidly inactivated by O_2_, diazotrophic cyanobacteria either fix N_2_ at night (to avoid photosynthetically evolved oxygen) or differentiate a thick-walled, photosystem II-deficient heterocystous cell type to specifically sustain daytime N_2_ fixation (Kumar *et al*., 2010). Members of the genus *Trichodesmium* fix N_2_ exclusively in the light (Dugdale *et al*., 1961; Capone *et al*., 1997), although the genus is affiliated to Section III filamentous cyanobacteria that are unable to form heterocysts and therefore expected to fix N_2_ (primarily) during the dark phase (see Bergman *et al*., 1997). Knowledge has expanded dramatically in regard to the diazotrophic physiology and molecular biology of *Trichodesmium*, but there are still gaps related to its unique cell biology and overall behavior in an ecophysiological context. We here summarize our current knowledge by highlighting its diazotrophic peculiarities from various perspectives.

## Speciation

*Trichodesmium erythraeum* was named by Ehrenberg in 1830 after observing blooms that discolored the water at the Bay of Tor in the Red Sea (Ehrenberg, 1830). Jules Verne (1869) in'20 000 leagues under the sea' also mentions blooms in this Bay (Box 1). Two other species, *T. thiebautii* and *T. hildebrandtii*, were named by Gomont (1892), and Wille (1904) later described another three species, *T. contortum, T. tenue*, and *T. radians*. These species were re-examined in 1995 (Janson *et al*., 1995) using specimens from the Indian Ocean, Caribbean, and Sargasso Seas. Ultrastructural arrangement of gas vesicles and glycogen clusters (carbon storage) were used as primary markers and separated the species into two clades: (i) *T. tenue* and *T. erythraeum* and (ii) *T. thiebautii*, *T. hildebrandtii*, and *T. contortum*. In 2001, a close relationship between *Trichodesmium* spp. (Lundgren *et al*., 2001) and marine cyanobacterial members of the genus *Katagnymene* (*K. pelagica* and *K. spiralis*; Lemmermann, 1900) was discovered using phylogenetic analysis of *nifH* gene sequences. Using the more variable fragment of the *hetR* gene, and a few other genetic markers, as targets revealed that the two *Katagnymene* species in fact cluster within one of the two *Trichodesmium* clades (Orcutt *et al*., 2002; Lundgren *et al*., 2005). Despite the morphological differences, *K. pelagica* and *K. spiralis* were, in addition, found to be the same species (Lundgren *et al*., 2005). Examining 21 cultivated isolates of *Trichodesmium/Katagnymene*, using genetic and morphological markers, Hynes *et al*. (2012) verified the existence of two *Trichodesmium* clades and suggested that these may inhabit different ecological niches, based on different pigment characteristics.

**Box 1**

From Jules Verne, ‘20,000 leagues under the sea’ [translated by Lewis Mercier, (Verne, 1872)]:

‘Here it is, M. Aronnax. According to my idea, we must see in this appellation of the Red Sea a translation of the Hebrew word 'Edom’; and if the ancients gave it that name, it was on account of the particular colour of its waters.' ‘But up to this time I have seen nothing but transparent waves and without any particular colour.’ ‘Very likely; but as we advance to the bottom of the gulf, you will see this singular appearance. I remember seeing the Bay of Tor entirely red, like a sea of blood.’ ‘And you attribute this colour to the presence of a microscopic seaweed?’ ‘Yes.’ ‘So, Captain Nemo, it is not the first time you have overrun the Red Sea on board the Nautilus?’ ‘No, sir.’

However, a full revision of the genera *Trichodesmium* and *Katagnymene* is now warranted, as the latter also includes several freshwater species (*T. iwanoffianum* Nygaard, *T. lacustre* Klebahn, *K. accurata* Geitler, *K. mucigera* Compére and *K. spirulinoides* An; see Komárek & Anagnostidis, 2005), one of which, in addition, represents the ‘type strain’ of the genus *Katagnymene*. Sequencing of additional genomes within the *Trichodesmuim* genus is also needed if we are to fully comprehend the taxonomy and phylogeny of the genus.

## An expanding and flexible genome

**Fig. 1:**
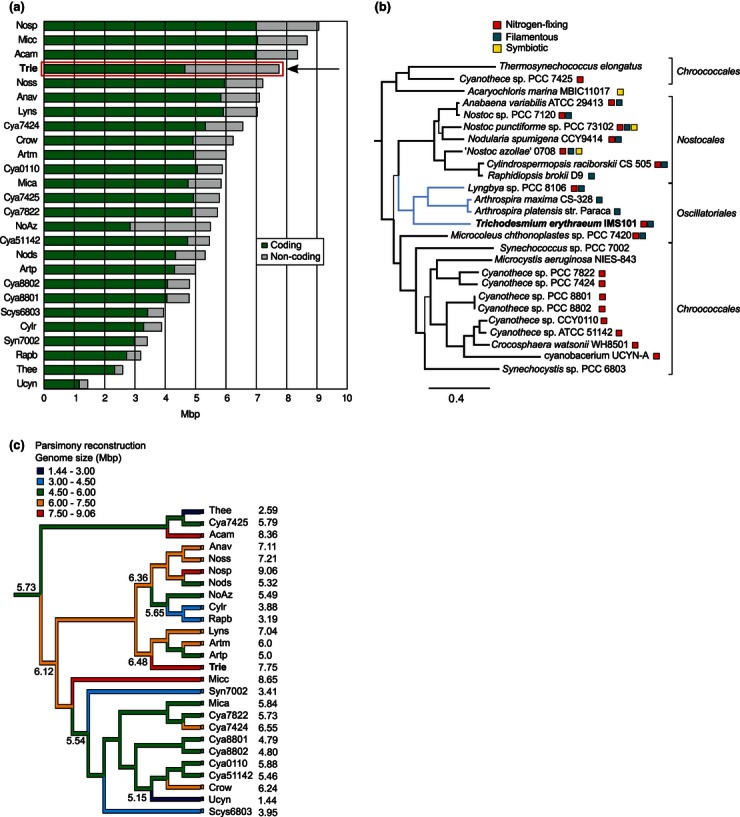
Phylogeny and genome properties of *Trichodesmium* IMS101. (a) Genome sizes and proportions of coding and noncoding nucleotides in genomes of organisms included in (b) and (c). Genomes are sorted by total size. The genome of *Trichodesmium* IMS101 is indicated by an arrow. (b) Maximum-likelihood phylogenetic tree based on a concatenated alignment of 285 single-copy orthologs. The tree is a subtree of a larger phylogeny of 58 cyanobacteria (see Larsson *et al*., 2011). Specific phenotypes for cyanobacteria are shown by the colored boxes next to the tip labels. The clade containing *Trichodesmium* (order *Oscillatoriales*) is highlighted with blue branches. Thick and thin branches indicate bootstrap support values (200 replicates) of 100 and between 58 and 84, respectively. Bar, 0.4 expected substitutions per site. (c) Ancestral genome sizes (reconstructed by parsimony) in the phylogeny from b. Organism names are abbreviated (see below for full names). Contemporary genome sizes (Mbp) are shown in the right margin and at specific nodes in the tree. Organism abbreviations are as follows: Acam = *Acaryochloris marina* MBIC11017, Anav = *Anabaena variabilis* ATCC29413, Artm = *Arthrospira maxima* CS328, Artp = *Arthrospira platensis* str. Paraca, Crow = *Crocosphaera watsonii* WH8501, Cya0110 = *Cyanothece* sp. CCY0110, Cya51142 = *Cyanothece* sp. ATCC51142, Cya7424 = *Cyanothece* sp. PCC7424, Cya7425 = *Cyanothece* sp. PCC7425, Cya7822 = *Cyanothece* sp. PCC7822, Cya8801 = *Cyanothece* sp. PCC8801, Cya8802 = *Cyanothece* sp. PCC 8802, Cylr = *Cylindrospermopsis raciborskii* CS505, Lyns = *Lyngbya* sp. PCC 8106, Mica = *Microcystis aeruginosa* NIES 843, Micc = *Microcoleus chthonoplastes* PCC7420, NoAz = ‘*Nostoc azollae*’ 0708, Nods = *Nodularia spumigena* CCY9414, Nosp = *Nostoc punctiforme* PCC73102, Noss = *Nostoc* sp. PCC7120, Rapb = *Raphidiopsis brookii* D9, Scys6803 = *Synechocystis* sp. PCC6803, Syn7002 = *Synechococcus* sp. PCC7002, Thee = *Thermosynechococcus elongatus*, Trie = *Trichodesmium erythraeum* IMS101, Ucyn = cyanobacterium UCYN-A. The figures are adapted from Larsson *et al*. (2011) with the author's permission.

*Trichodesmium erythraeum* IMS101 (from now on *Trichodesmium* IMS101) was one of the first strains isolated into axenic cultures (Prufert-Bebout *et al*., 1993) and still represents the only sequenced genome within the genus (http://genome.jgi-psf.org/trier/trier.home.html). The genome, which comprises 7.75 Mbp, is among the larger cyanobacterial genomes sequenced to date (Fig. 1a). A recent phylogenetic survey of 58 sequenced cyanobacterial genomes, based on a concatenated alignment of 285 protein orthologs, verified that *Trichodesmium* IMS101 is affiliated to a lineage composed of other filamentous nonheterocystous species within *Oscillatoriales* (Fig. 1b; Larsson *et al*., 2011): the marine *Lyngbya* sp. PCC 8106 and two species within *Arthrospira* (*A. platensis* and *A. maxima*; previously denoted *Spirulina*). These are all ecologically successful and widespread inhabitants of marine waters and alkaline lakes, respectively. Recent analyses based on the 16S rRNA gene sequence give a similar clustering of *Trichodesmium* IMS101 (Schirrmeister *et al*., 2011). Larsson *et al*. (2011) also showed that the capacity to fix N_2_ within this *Trichodesmium* clade was lost in *A. platensis* and *A. maxima*, as also verified by Latysheva *et al*. (2012), while retained in the deeper-branching *Trichodesmium* and *Lyngbya* sp. PCC 8106. Interestingly, these four lineages all possess *hetR* (Larsson *et al*., 2011), a gene encoding a protease with a key function in cell differentiation (N_2_-fixing heterocysts and resting akinetes; see Kumar *et al*., 2010), although they all lack these developmental capacities. Additionally, comparative genomic analyses show that several other gene orthologs involved in heterocyst differentiation are present in the *Trichodesmium* IMS101 genome (e.g. *hetCF, patB*) while others, not unexpectedly, are missing such as those involved in the deposition of the heterocyst outer envelope (e.g. *hglCDE, hepB*) (Table S1; El-Shehawy *et al*., 2003; Larsson *et al*., 2011). However, orthologous genes are not always functionally equivalent. For instance, the *sepJ* gene of *Trichodesmium* is missing a vital domain essential for filament integrity under nitrogen deprivation, although it fully complements a *sepJ* deletion mutant of *Nostoc* sp. PCC7120 when grown in the presence of combined nitrogen (Mariscal *et al*., 2011). The *nif* gene operon of *Trichodesmium* IMS101 is conserved in a manner typical of some heterocystous cyanobacteria, although *Trichodesmium* lacks the large DNA insertion element present in the structural *nifD* gene of several of the heterocystous cyanobacteria, as well as the intergenic region between *nifB* and *nifVZT*/*cysE* (Fig. S1). These findings strengthen an evolutionary relationship between the genus *Trichodesmium* and the heterocystous clade, whereas distinct differences are also apparent, relationships now worth examining in greater detail.

Another notable feature of the *Trichodesmium* IMS101 genome is its comparatively low coding capacity (Larsson *et al*., 2011). With *c*. 40% noncoding DNA, it holds one of the lowest coding percentages among all to date sequenced genomes of cyanobacteria (Fig. 1a) and other bacteria (Hou & Lin, 2009). The intergenic sequences within the *Trichodesmium* IMS101 genome (459-bp median length) are also relatively large for cyanobacteria (14.5- to 231-bp median intergenic length in 39 other finished cyanobacterial genomes). The reason for these large and presumably noncoding intergenic spacers in the *Trichodesmium* IMS101 genome is unknown. It is, however, interesting to note that among the 58 genomes examined, the genome of *Trichodesmium* IMS101 is one of a few in which the genome is currently expanding in size, as is also the case for the genomes of the limnic *Microcystis aeruginosa* NIES 843 and the marine *Acaryochloris marina* MBIC11017 (Fig. 1c; Larsson *et al*., 2011). This suggests that the genome of *Trichodesmium* IMS101 is in an expanding dynamic state, in contrast to the shrinking genomes of the unicellular marine genera *Synechococcus* and *Prochlorococcus* (with genomes < 2 Mbp; Palenik *et al*., 2006; Kettler *et al*., 2007), genera which to a large extent share the same tropical/subtropical marine habitat as *Trichodesmium*. Based on these data, it is suggested that different strategies are used to cope with the various constraints enforced by these oligotrophic oceans (Larsson *et al*., 2011). *Trichodesmium* may use a strategy to flexibly adapt by incorporating functions and capacities when needed (e.g. via horizontal gene transfer, HGT) and maintain gene duplications (in-paralogs) affecting *c*. 10% of all genes in the *Trichodesmium* IMS101 genome, as a mechanism to promote genome expansion and organismal adaptations (Swingley *et al*., 2008; Treangen & Rocha, 2011). One example of HGT in *Trichodesmium* IMS101 is the acquisition of long eukaryotic triglyceride collagen protein fibers that may sustain *Trichodesmium* colony formation (Layton *et al*., 2008). Paralogs and horizontally gained genes that do not provide a fitness advantage will undergo inactivation (sequence divergence and loss of function) and eventually be lost. Indeed, 21% of in-paralogs in *Trichodesmium* IMS101 appear to have been subject to inactivation and are now present only as pseudogenes within the genome (Larsson *et al*., 2011), thereby contributing to the abundance of noncoding nucleotides (Fig. 1a). Considering that bacterial genomes are subject to a deletion bias (Mira *et al*., 2001), the large noncoding proportion of the *Trichodesmium* IMS101 genome which cannot be attributed to remnants of previously functional genes (pseudogenes) is enigmatic. However, it is possible that at least parts of these intergenic regions contain as yet nonannotated genes or small RNAs (Hewson *et al*., 2009; Shi *et al*., 2009). It appears that the unicellular marine cyanobacteria with reduced genomes (e.g. *Prochlorococcus* spp. in Clade II) use an opposite strategy to compete for life-space and survival, that is maintain a large surface-to-volume ratio and fewer genes, a strategy recently characterized as ‘cryptic escape’ (Yooseph *et al*., 2010).

## The diazocytes – separation in space

**Fig. 2:**
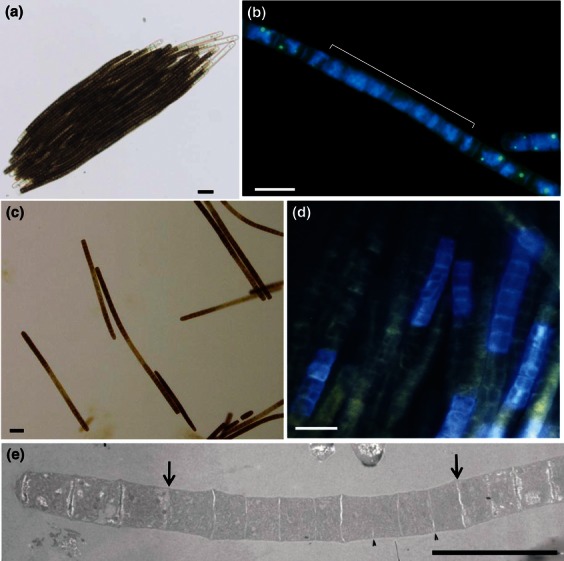
Morphological characteristics of *Trichodesmium* trichomes, with emphasis on cell differentiation and their nitrogenase containing cell type, the diazocytes. (a) A light micrograph depicting a dark pigmented colony consisting of longitudinally arranged trichomes of a newly isolated strain, *T. erythraeum* TNZ0801. Scale bar, 25 μm. (b) The DNA distribution in cells of a *Trichodesmium* IMS101 trichome visualized after staining with the dye 4′,6-diamidino-2-phenylindole (DAPI), fluorescing blue. Note the DNA presence in all cells, the centrally located diazocyte-like zone (marked) being recognized as they are devoid of the yellow/green fluorescent granules representing polyphosphate storage. Scale bar, 20 μm. (c) Trichomes of *Trichodesmium* IMS101 stained with Lugol's solution. Note several lighter-stained central diazocyte zones, in which catabolic carbon metabolism has degraded the Lugol-stainable stored carbon supplies. Scale bar 20 μm. (d) Fluorescence *in situ* immunolocalization of NifH into groups of adjacent cells, diazocytes, in central areas of intact trichomes of *Trichodesmium* IMS101. The NifH protein is detected as a blue fluorescence due to a secondary anti-NifH-antibody coupled to a blue-fluorescing chromophore. Scale bar, 10 μm. (e) Transmission electron micrograph depicting a longitudinally sectioned trichome of *Trichodesmium* IMS101. Note the more homogenous zone of cells, representing diazocytes between the arrows. Arrowheads point to ongoing cell division (the formation of division septa) in two of the diazocytes. Scale bar, 20 μm.

A reduced oxygen environment is a prerequisite for effective N_2_ fixation activity in *Trichodesmium* as in all other bacteria. Both transcription of *nif* genes and biosynthesis of the nitrogenase enzyme complex have been found to be sensitive to oxygen inactivation (Zehr *et al*., 1997; Staal *et al*., 2007). Ever since the pioneering studies by Dugdale and co-workers (Dugdale *et al*., 1961), a compelling research area has therefore been to elucidate how *Trichodesmium* reconciles oxygenic photosynthesis and oxygenophobic N_2_ fixation within its ‘heterocyst-free’ physiology. Initially, N_2_ fixation was proposed to be limited to low-oxygen or anaerobic regions in the center of *Trichodesmium* colonies (Paerl & Bebaut, 1988). However, later, it became apparent that colony formation was not a prerequisite as ‘free’ trichomes, the dominant form in *Trichodesmium* laboratory cultures, are also able to fix N_2_. Attention then switched to the structural differences along the *Trichodesmium* trichomes that early on were observed using light microscopy, recognized as ‘nongranulated’ or ‘lighter’ cell regions in parts of the trichomes (Carpenter & Price, 1976; Bryceson & Fay, 1981; Li & Lee, 1990), in both single trichomes, and colony-associated trichomes. This cellular arrangement occurs in both natural populations and cultures of *Trichodesmium* IMS101 (Lin *et al*., 1998; El-Shehawy *et al*., 2003). On average, ∼ 15% of the total cell population of the trichomes may be described as less granulated or lighter (more transparent). These cells are arranged in strings or ‘zones’ composed of ∼ 2–30 cells, but are not always obvious in LM (Fig. 2a). However, when stained (e.g. with Lugol's solution; Fig. 2c), each trichome harbors typically 1–2 such zones per trichome, but up to four zones have been observed in longer trichomes (El-Shehawy *et al*., 2003). The nongranulated appearance is caused by a diminished number and/or size of subcellular structures such as cyanophycin granules, gas vacuoles, and polyphosphate granules (Fig. 2c and e), while additional membranes are synthesized (Fredriksson & Bergman, 1997).

The ‘lighter’ cells were in 1991 (Bergman & Carpenter, 1991) proven to be the nitrogenase enzyme containing cells in *Trichodesmium* (Fig. 2d). The existence of a ‘spatial’ nitrogenase sequestration mechanism in a nonheterocystous cyanobacterium was thereby proven. The cells were subsequently termed diazocytes: *di* (two) *azo* (nitrogen) *cyte* (cell) (Fredriksson & Bergman, 1997). The exclusive localization of nitrogenase in diazocytes was corroborated by immuno-TEM (sectioned trichomes) and immuno-LM (whole-mount intact trichomes; Fig. 2d) analyses of both cultured and natural populations from the Indian, Pacific, and Atlantic Oceans, using a battery of antibodies (Bergman & Carpenter, 1991; Bergman *et al*., 1993; Janson *et al*., 1994; Fredriksson & Bergman, 1997; Berman-Frank *et al*., 2001b), including also one monoclonal anti-*Trichodesmium* IMS101-NifH antibody (targeting the smaller Fe protein subunit; Zehr *et al*., 1990; Bergman *et al*., 1993). The frequency of the diazocytes is lower at dawn and increased toward noon and is negatively regulated by the presence of combined nitrogen (Fredriksson & Bergman, 1995; Lin *et al*., 1998; Sandh *et al*., 2009, 11). Indeed, theoretical models suggest that a spatial separation of processes (such as nitrogen fixation and photosynthesis) favors biomass production compared to temporal separation (Rosetti & Bagheri, 2012) as, for instance, in unicellular cyanobacteria (Bergman *et al*., 1997). As a few other studies have suggested that the nitrogenase enzyme is present in all cells within the trichomes (Paerl *et al*., 1989; Ohki, 2008; Orcutt *et al*., 2009), a variation in cellular localization may exist depending on different environmental conditions or species examined. Using ^15^N and Nano-SIMS, Finzi-Hart *et al*. (2009) observed that the fixed N is rapidly distributed into the majority of cells along *Trichodesmium* trichomes, although cells in the center showed a lower ^15^N label, a pattern that may suggest a zone of diazocytes. A lower ^15^N label is also typical for heterocysts analyzed by Nano-SIMS due to a most rapid transfer of fixed nitrogen out of these cells (Popa *et al*., 2007; Ploug *et al*., 2010).

**Fig. 3:**
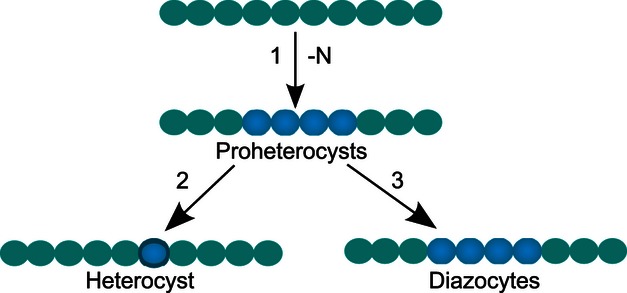
A potential evolutionary scenarium for the development of the diazocytic phenotype in *Trichodesmium*. (1) An ancient nonheterocystous filamentous cyanobacterium, a forerunner of Nostocales and Oscillatoriales (Fig. 1b and c), under nitrogen deprivation conditions develop strings of proheterocysts (as in *Anabaena*-type spp.; Wilcox *et al*., 1975). (2) The majority of the proheterocysts revert back into vegetative cells, while one continus the evolution into a thick-walled heterocyst, the dominating nitrogen-fixing phenotype in limnic and terrestrial ecosystems today. (3) In oceans, this proheterocystous phenotype may have been retained and evolved into the strings of diazocytes we see in *Trichodesmium* spp. today, while the closest relatives (Fig. 1b) either fix nitrogen in the dark (*Lyngbya* spp.; e.g. Lundgren *et al*., 2003) or have lost the capacity to fix nitrogen (*Arthrospira* spp.; Larsson *et al*., 2011; Latysheva *et al*., 2012).

It was recently shown that, as for the differentiation of heterocysts, removal of combined nitrogen from the growth medium is the sole and sufficient mean needed to elicit the development of the centrally located nitrogenase containing diazocytes (Fig. 2d) in *Trichodesmium* IMS101 (Sandh *et al*., 2012). The fact that the development of diazocytes takes between 8–27 h and that changes in cellular ultrastructure precede the expression of the nitrogenase enzyme (Sandh *et al*., 2012) strongly argues for a genetically based developmental background. Phylogenetically, the nonheterocystous *Trichodesmium* clade is a sister group to the heterocystous clade (Fig. 1b) and *Trichodesmium* shares several genomic and behavioral features with this clade (see above, below and Table S1). For instance, some heterocystous cyanobacteria (*Anabaena* sp.) first develop strings or subsets of adjacent proheterocysts upon nitrogen deprivation, while only the central cell develops into a mature heterocyst and the other regresses into vegetative cells (Wilcox *et al*., 1975). The similarity of the patterning of proheterocysts and diazocytes hints that several early regulatory elements may be shared in the nitrogen-regulated pathways of heterocystous genera and *Trichodesmium*. Diazocytes may during evolution have ‘frozen’ at this more minimalistic initial stage (Fig. 3), as a full differentiation is disadvantageous for the conditions offered in oceans (Staal *et al*., 2003; : Stal, 2009). In contrast to heterocysts, the diazocytes are not terminally differentiated cells and retain their ability to divide (Fig. 2e; Fredriksson & Bergman, 1995), which may also contribute to their more flexible life style and allow the diazocytes (*Trichodesmium*) to more easily/rapidly adapt to prevailing conditions. To what extent *Trichodesmium* and heterocystous cyanobacteria share additional regulatory mechanisms that govern nitrogen deprivation signaling and pattern formation is now of great interest to be resolved.

## Diazotrophy – separation in time

The timing of the N_2_ fixation physiology of *Trichodesmium* is governed by the circadian clock (Chen *et al*., 1996, 1998; Dong & Golden, 2008). This regulates the transcription of the *nif* genes, the daily *de novo* synthesis of the subunits of the nitrogenase enzyme (NifHDK), a post-translational modification of NifH (Capone *et al*., 1990; Zehr *et al*., 1993; Chen *et al*., 1996), and the supply of appropriate levels of energy and reducing equivalents necessary for N_2_ fixation activity (Staal *et al*., 2007).

Hence, it appears that *Trichodesmium* spp. not only separates N_2_ fixation physically from net oxygen evolution via the development of a special cell type (diazocytes), although this may be the major protective mechanism, but also separates these two incompatible processes temporarily (Berman-Frank *et al*., 2001b), although in a more subtle way than in other nonheterocystous cyanobacteria (Bergman *et al*., 1997). For instance, in contrast to in the latter and in concert with heterocystous cyanobacteria, nitrogenase activity in *Trichodesmium* operates *within* the day/light phase of the diel cycle, however, at a period around mid-day when the oxygen production is lowered and oxygen-scavenging mechanisms enhanced (respiration/Mehler reaction). This ‘mid-day depression’ in photosynthetic oxygen evolution is manifested as a lower quantum yield (∼ 50%) and a low or negative net O_2_ evolution (Berman-Frank *et al*., 2001b). Chlorophyll fluorescence kinetic microscopy at the single-cell level has also revealed flexible temporal and spatial switching between high fluorescence states (within a row of cells) and recovery states during subsequent non-N_2_-fixing periods in *Trichodesmium* IMS101 (Küpper *et al*., 2004). This rapid sequential switching within type I cells (functional PSII activity and enhanced Mehler reaction; potentially being the diazocytes) would allow diazotrophy even in cells lacking thick cell walls, may be orchestrated by rearrangements of the phycobilisomes between PSI and PSII (Küpper *et al*., 2009; Andresen *et al*., 2010), and may constitute the part of *Trichodesmium*'s nitrogenase-protecting mechanism.

The increase in Mehler reaction (Kana, 1993; Milligan *et al*., 2007) and dark respiratory activity during the diazotrophic periods (Carpenter & Roenneberg, 1995; Kranz *et al*., 2009) is supported by the enhanced levels of the respiratory enzyme cytochrome c oxidase detected using proteomic analyses on N_2_-fixing cultures of *Trichodesmium* IMS101 (Sandh *et al*., 2011) and the enhanced levels of this enzyme specifically in the diazocytes, detected using immunogold localization in natural *Trichodesmium* populations (Bergman *et al*., 1993). In addition, Bryceson & Fay (1981) described zones of cells with higher tetrazolium salt depositions within specific areas of the trichomes, which support a reducing environment within the diazocytes. Although the cellular localization is not yet resolved, recent reports on novel carotenoids with strong antioxidant activity in *Trichodesmium* (Kelman *et al*., 2009) are also interesting in this context and worth examining further.

In addition to a lower net oxygen evolution, the CO_2_ fixation is also lowered at mid-day (Berman-Frank *et al*., 2001b) as recently verified using Nano-SIMS (Finzi-Hart *et al*., 2009). Earlier ^14^C labeling experiments also demonstrated a lower CO_2_ incorporation in central parts of the trichomes (Paerl, 1996). The decreased CO_2_ fixation impacts the subcellular storage of carbon (glycogen) and the general carbon metabolism. For instance, carbohydrate storages are degrading (Fig. 2c; El-Shehawy *et al*., 2003; Sandh *et al*., 2011), and recent proteomic analysis verifies a shift toward a catabolic carbon metabolism under diazotrophy, that is, a down-regulation of enzymes involved in glycogen storage and an up-regulation of enzymes involved in central carbon metabolism (Sandh *et al*., 2011). Diazotrophic conditions also led to enhanced levels of proteins involved in the biosynthesis of reducing equivalents (NADPH; through the oxidative pentose phosphate pathway) and the generation of a micro-oxic environment, consistent with increased respiratory activities (Sandh *et al*., 2011) and oxygen levels at mid-day.

*Trichodesmium* IMS101 also practices a light/dark (day/night) separation of other basic cellular processes. While the highly energy-demanding processes, such as CO_2_ fixation and diazotrophy, take place in light/day phase, cell division and diazocyte development are more pronounced in the dark/night phase (Chen *et al*., 1999; Sandh *et al*., 2009). Temporal separation of similar processes on a diel basis has previously been observed in marine unicellular cyanobacteria (Holtzendorff *et al*., 2001, 2002; Stockel *et al*., 2008; Shi *et al.,* 2010). The circadian clock governs many of these processes and is entrained by the cellular ATP/ADP ratio, which in turn is governed by photosynthesis (Rust *et al*., 2011). Taken together, current data suggest that besides the development of diazocytes, some more subtle physiological mechanisms may act in concert to optimize diazotrophy in *Trichodesmium*.

## Adaptation to nutrient stress

The nitrogen fixed in *Trichodesmium* is, as in other cyanobacteria, assimilated via the glutamine synthetase–glutamate synthase (GS-GOGAT) pathway, and as in heterocysts, the GS protein levels are higher in the diazocytes (Carpenter *et al*., 1992) to prevent feedback inhibition of the nitrogenase activity by the accumulation of the ammonia produced. Likewise, externally administrated sources of nitrogen negatively affect the expression of the *nif* genes, the synthesis of the nitrogenase enzyme, the nitrogenase activity, and diazocyte abundance in *Trichodesmium* (Ohki *et al*., 1991; Lin *et al*., 1998; Mulholland *et al*., 2001; El-Shehawy *et al*., 2003; Holl & Montoya, 2005; Sandh *et al*., 2011). As in other cyanobacteria (Herrero *et al*., 2004), when subject to N deprivation, there is a significant upshift in the cellular C : N ratio in *Trichodesmium* (Kranz *et al*., 2009), which may be a signal for enhanced transcription by the transcription factor NtcA (Table S1) of N-regulated genes. However, *Trichodesmium* appears to be flexible in this context, being able to fix N_2_ in the presence of low concentrations of dissolved inorganic and organic nitrogen (Holl & Montoya, 2005), and the transcript of *ntcA* is not exclusively regulated by the availability of, for example, ammonium (Post *et al*., 2012), which suggests that our knowledge in this area is still limited.

Phosphorus and iron are critical nutrients restricting growth and N_2_ fixation in today's oceans (Sanudo-Wilhelmy *et al*., 2001; Mills *et al*., 2004; Sohm *et al*., 2008; Moore *et al*., 2009). To overcome P limitations in oligotrophic waters, the *Trichodesmium* colonies migrate vertically in the water column to scavenge P and other nutrients using a buoyancy-regulating mechanism. This is provided by the pronounced gas vacuoles of *Trichodesmium*, which can withstand pressures down to depth of about 100–200 m (the highest known; Kromkamp & Walsby, 1992). In the upper euphotic zone, the colonies capture and store carbon and nitrogen (as glycogen and cyanophycin granules; Romans *et al*., 1994), and with this ‘ballast’, the colonies sink into deeper waters where P species may be acquired (Romans *et al*., 1994; Villareal & Carpenter, 2003; White *et al*., 2006b; Hewson *et al*., 2009). As the cellular ballast is metabolized in the deeper darker waters, the subsequently lighter colonies return to the euphotic zone to again capture light energy.

*Trichodesmium* is also known to adjust to periods of low P bioavailability by adopting high cellular N : P ratios (White *et al*., 2006a) in part by a substitution of its phospholipids by non-P membrane lipids (Van Mooy *et al*., 2009). Phosphorus uptake is also maximized via the uptake of both inorganic and organic phosphorous species (Stihl *et al*., 2001; Fu *et al*., 2005; Dyhrman *et al*., 2006; Orchard *et al*., 2009; Beversdorf *et al*., 2010; White *et al*., 2010). Enzymes hydrolyzing phosphoesters (alkaline phosphatase; Stihl *et al*., 2001; Orchard *et al*., 2009) and phosphonates (e.g. phosphonate hydrolase; Dyhrman *et al*., 2006) to yield phosphate have been identified in *Trichodesmium*. However, the globally widespread *T. thiebautii* lacks one of the alkaline phosphatase–encoding genes, *phoA* (Orchard *et al*., 2003). It has been shown that colonial alkaline phosphatase activities in *Trichodesmium* may rather be further enhanced by quorum-sensing signals (acylated homoserine lactones) released from colony-associated microorganisms (Van Mooy *et al*., 2012). The various phosphate pools available can be utilized either individually or in combination to sustain growth and N_2_ fixation (White *et al*., 2010). Any ‘luxury’ uptake of phosphate is stored in subcellular structures (polyphosphate granules), which are common in natural *Trichodesmium* populations (Romans *et al*., 1994). As increased polyphosphate storages have been found in phosphate-starved cells (Orchard *et al*., 2010), the regulation of these subcellular structures in *Trichodesmium* is enigmatic. The variability among *Trichodesmium* species in relation to P-uptake genes (Orchard *et al*., 2003) also raises the question of niche differentiation and calls for further investigation.

Iron is a pivotal cofactor in a number of cellular processes, such as photosynthesis, N_2_ fixation (in nitrogenase), and oxygen scavenging (Kustka *et al*., 2003; Shi *et al*., 2007). The high iron content of *Trichodesmium* cells suggests an efficient uptake and detainment capacity of iron and may even make *Trichodesmium* colonies ecologically valuable sources of this otherwise poorly soluble element in oligotrophic oceans (Kustka *et al*., 2003; Whittaker *et al*., 2011). No obvious genes in the *Trichodesmium* genome seem to encode for siderophores (Chappell & Webb, 2010), while the genes for transporters of siderophore-bound Fe^3+^ and Fe^2+^ and for enzymes related to cellular storage of iron are present (Castruita *et al*., 2006; Chappell & Webb, 2010). However, the involvement of siderophores in enhancing the uptake of low iron concentrations in *Trichodesmium* colonies has been reported (Achilles *et al*., 2003). The current hypothesis is that these siderophores are synthesized by associated microorganisms that indirectly facilitate the uptake of iron by *Trichodesmium* (Achilles *et al*., 2003). On the other hand, a recent study showed that such siderophore-bound iron is rather consumed by the bacteria than by the *Trichodesmium* cells (Roe *et al*., 2011). Another option is that the *Trichodesmium* colony formation *per se* facilitates the capture of enough particulate iron from, for example, eolian dust depositions to feed the colonies (Rubin *et al*., 2011).

Iron depletion is known to lead to a decrease in the frequency of diazocytes (Berman-Frank *et al*., 2001a; Küpper *et al*., 2008) and to a down-regulation of N_2_ fixation, while photosynthetic capacities are maintained (Shi *et al*., 2007; Brown *et al*., 2008; Küpper *et al*., 2008). Iron limitation may also elicit a switch in the phycobiliproteins being used (Küpper *et al*., 2008). Other cyanobacteria have also been shown to replace ferredoxin with the iron-free flavodoxin (Sandmann *et al*., 1990). Monitoring *isiB* transcription, encoding a flavodoxin, has suggested that *Trichodesmium* populations in the Atlantic Ocean are rarely, or not at all, iron limited, while those in the Pacific Ocean are (Chappell *et al*., 2012). However, the expression of the two *fld* genes, encoding flavodoxins, in *Trichodesmium* IMS101 is also regulated by N availability and growth stage (Lin *et al*., 2009; Chappell & Webb, 2010; Sandh *et al*., 2011), and their proposed use as iron limitation ‘markers’ may be questioned. Rather, the enhanced *idiA* transcription and IdiA levels noted under iron limitation may be a more suitable marker for iron stress in *Trichodesmium* (Webb *et al*., 2001; Chappell & Webb, 2010). As a strong up-regulation of Dps, yet another protein related to iron acquisition, was observed on the transfer of *Trichodesmium* to diazotrophic conditions (N stress), the role of Dps and other proteins related to iron acquisition and metabolism now also needs attention.

## Impact on the ecosystem

The *Trichodesmium* abundance is roughly limited to waters warmer than 20 °C, and temperature tolerance for growth and N_2_ fixation in cultured strains of *Trichodesmium* (*T. erythraeum* IMS101, *T. erythraeum* GBRTRLI101 and *T. tenue* H94) ranges from 20 to 34 °C, with optimal temperatures being 24–30 °C depending on species and other growth conditions (Breitbarth *et al*., 2007; Chappell & Webb, 2010). However, *Trichodesmium*-like cyanobacteria (*nifH* and *hetR* phylogenies) were recently reported in Arctic waters suggesting wider temperature limits (Díez *et al*., 2012). Indeed, the very spotty nature of surface blooms of *Trichodesmium* does not represent the entire population in the ecosystem monitored and illustrates the difficulty in estimating the full global distribution of *Trichodesmium* via bloom registrations. In spite of this limitation, monitoring such blooms via remote sensing (via the SeaWiFS satellite; Subramaniam *et al*., 2002) verified that *Trichodesmium* blooms occur roughly between 20˚N and 20˚S in the eastern Pacific Ocean and that patches may occur even toward 40˚N and 40˚S in the Atlantic and the western Pacific and Indian Oceans (1998 and 2003; Westberry & Siegel, 2006).

Besides *Trichodesmium*, numerous unicellular cyanobacteria share the same marine aquatic environment, notably the small-celled non-N_2_-fixing genera *Prochlorococcus* and *Synechococcus* (cell diameter ∼ 1 μm, genome sizes of ∼ 2 Mbp; Partensky *et al*., 1999; Scanlan *et al*., 2009), but also several N_2_-fixing unicellular cyanobacteria (Zehr *et al*., 2001; Montoya *et al*., 2004; Moisander *et al*., 2010). Among the latter are representatives of the marine genera *Cyanothece*, *Crocosphaera*, and N_2_-fixing cyanobacteria of the ‘group A’ *nifH* phylotype (e.g. UCYN-A). These diazotrophic unicellular cyanobacteria may show a broader temperature tolerance (15–30 °C) than *Trichodesmium*, and some have been recovered from waters with detectable nitrate concentrations (Langlois *et al*., 2005). Observed community shifts from filamentous cyanobacteria in surface waters to unicellular cyanobacteria and/or heterotrophic bacteria in deeper waters (Langlois *et al*., 2005) may also suggest different ecological niche occupancies. Although estimates of the relative contribution to the total biogenic N_2_ fixation in oceans by unicellular cyanobacteria (and heterotrophic bacteria) are increasing (Halm *et al*., 2012; Sohm *et al*., 2011a,b; Turk *et al*., 2011; Zehr & Kudela, 2011), the role of *Trichodesmium* as a C and N source in the world's oceans is still profound. For instance, *Trichodesmium* may account for up to 50% of the *nifH* genes in the North Atlantic Ocean (0°N – 42°N and 67°W – 13°W; Langlois *et al*., 2008), and *Trichodesmium/Katagnymene* represent up to 10^6^ *nifH* genes per liter, while the unicellular cyanobacteria were represented by 10^5^ *nifH* genes per liter and proteobacteria by 10^4^ *nifH* genes per liter (Rijkenberg *et al*., 2011). Tyrrell *et al*. (2003) reported an even higher colony abundance of *Trichodesmium* in the tropical Atlantic Ocean (0–15^o^N and 20^o^W), and a *Trichodesmium* surface bloom covered about 100 000 km^2^ in the Arabian Sea (Capone *et al*., 1998).

For still unknown reasons, large segments of the *Trichodesmium* population are suddenly trapped at the surface forming easily observed pigmented layers of dying and decomposing cells (‘blooms’) (Capone *et al*., 1998). Such decomposing blooms function as gigantic ‘fertilizer heaps’ releasing large quantities of carbon, nitrogen, and other nutrients for the benefit of nondiazotrophic and heterotrophic biota in the surrounding water bodies. The cause of this destructive ‘bloom’ phenomenon is unknown, while the involvement of viral infections (Hewson *et al*., 2004) and/or autocatalyzed cell death processes (Berman-Frank *et al*., 2004) have been proposed, but the question is still open. The nitrogen fixed by *Trichodesmium* may, in addition, enter marine food webs via grazing by tunicates, copepods, and fish (Roman, 1978; Bryceson, 1980; Carpenter, 1983; Oneil & Roman, 1994; Eberl & Carpenter, 2007). Nitrogen isotope ratios in zooplankton in the North Atlantic Ocean strongly indicate that N_2_ fixation is a major source of nitrogen for the marine zooplankton community (Montoya *et al*., 2002). This is at the same time unexpected, as the *Trichodesmium* toxin production has been inferred as a predator-deterring function (Layton *et al*., 2008) as well as a cause of death of several eukaryotic organisms, notably the copepod *Acartia tonsa* (Guo & Tester, 1994), several species of fish (Endean *et al*., 1993), and pearl oysters (Negri *et al*., 2004). *Trichodesmium* is now known to release different secondary metabolites, such as toxins, including the lipophilic chlorinated trichotoxin (Schock *et al*., 2011), the palytoxin causing clupeotoxism in humans via fish (Kerbrat *et al*., 2011), and the neurotoxin β-N-methylamino-L-alanine (Cox *et al*., 2005), the latter also found in diazotrophic bloom-forming cyanobacteria in the Baltic Sea (Jonasson *et al*., 2010). The ecological function of these toxins is still unknown.

**Fig. 4:**
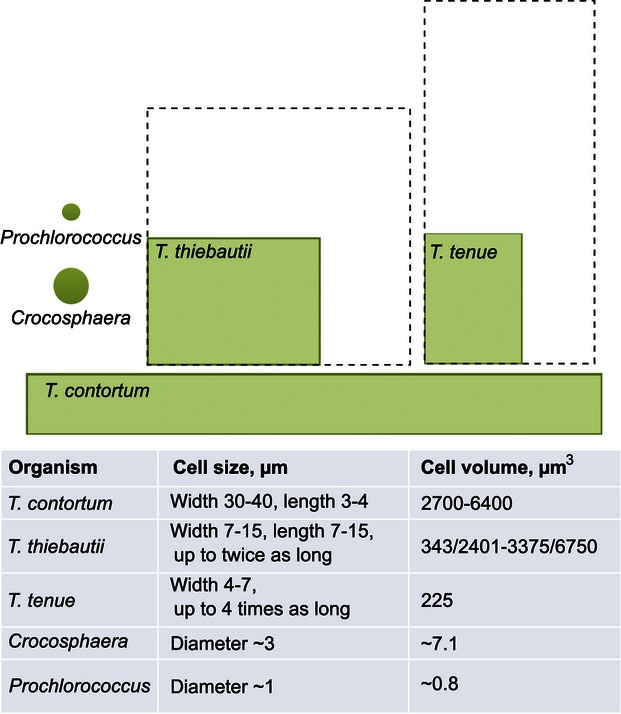
Schematic illustration of approximate cell size differences of *Trichodesmium* species and some unicellular cyanobacteria. The approximate cell sizes and volumes of different species of *Trichodesmium* are compared to the cell volumes of representatives of the nondiazotrophic but ubiquitous unicellular cyanobacterial genus *Prochlorococcus* and the unicellular diazotrophic genus *Croccosphaera*. Note the many-fold larger volume of the *Trichodesmium* cells. Hatched line shows maximum cell sizes. *Trichodesmium* cell sizes are according to Janson *et al*. (1995).

Yet another factor that may play a role besides the mere cell number of an organism is their cell size/cell volume. As illustrated in Fig. 4, the size of *Trichodesmium* cells (approximate sizes given) makes the cellular volumes of this organism ‘gigantic’ compared to, for instance, cells of the unicellular genus *Prochlorococcus* and the diazotrophic genus *Croccosphaera*. As about 10–20 cells in each *Trichodesmium* filament are diazocytes (filled with NifH; Fig. 2b) and a large fraction of the nitrogen fixed may be released (as dissolved organic nitrogen or ammonium) from actively growing *Trichodesmium* populations (Capone *et al*., 1994; Glibert & Bronk, 1994; Mulholland & Capone, 2001; Mulholland *et al*., 2004; Mulholland, 2007), each *Trichodesmium* cell and filament may be viewed as a highly important source of new nitrogen for all nondiazotrophic small cells (unicellular cyanobacteria and bacteria) when sharing similar N-depleted aquatic marine environments.

## *Trichodesmium* in future scenarios

The global importance of *Trichodesmium* in oceanic biogeochemistry has triggered numerous studies mimicking future global warming scenarios. As mentioned above, the optimum temperature range for growth and nitrogen fixation of *Trichodesmium* is 24–30 °C (Breitbarth *et al*., 2007; Chappell & Webb, 2010), but *Trichodesmium* also survives lower temperatures and darkness (White *et al*., 2006b; Breitbarth *et al*., 2007). The ability to live at lower temperatures and in darkness is essential for their vertical migrations (White *et al*., 2006b) and may explain the occurrence of *Trichodesmium* in temperate (see LaRoche & Breitbarth, 2005) and potentially in cooler (Díez *et al*., 2012) waters. Global warming will lead to increased stratification resulting in shallower mixed layers and increased irradiance. High light intensity (up to 1000 μE) stimulates growth, diazocyte abundance, and N_2_ fixation in *Trichodesmium* (Andresen *et al*., 2010; Kranz *et al*., 2010; Levitan *et al*., 2010) and provokes changes in pigment composition (Andresen *et al*., 2010), and correlates with increased O_2_ evolution and CO_2_ fixation (Kranz *et al*., 2010). Higher light intensities also cause faster protein turnover (Andresen *et al*., 2010) and an increase in RuBisC/O:PSII ratio (Brown *et al*., 2008). The down-regulation in O_2_ production via PSII correlates with the earlier noted peaks in N_2_ fixation at noon (Berman-Frank *et al*., 2001b). Increased pCO_2_ levels will not only stimulate CO_2_ fixation, but also stimulate N_2_ fixation (dependent on carbon skeletons for sequestration of the ammonium produced) and growth in *Trichodesmium* (Hutchins *et al*., 2007; Levitan *et al*., 2007, 2010; Ramos *et al*., 2007; Kranz *et al*., 2009, 2010). One mechanism may be energy relocation from the costly carbon-concentrating mechanism (CCM; Badger *et al*., 2006; Kranz *et al*., 2011) toward CO_2_ and N_2_ fixation (Levitan *et al*., 2007; Kranz *et al*., 2011). This may in turn increase the release of the newly fixed N into the surrounding water body, thereby enhancing primary production of other organisms (Hutchins *et al*., 2007). Hence, in a scenario of increased temperatures and CO_2_ concentrations in the world's oceans, the abundance of surface blooms of *Trichodesmium* is expected to increase (Breitbarth *et al*., 2007; Hutchins *et al*., 2007; Levitan *et al*., 2007), unless other unforeseen natural factors provoke the opposite reaction. For instance, element colimitations, rather than single-element limitations, may regulate the growth and N_2_ fixation of natural *Trichodesmium* populations (Mills *et al*., 2004; Hutchins *et al*., 2007). Also, Garcia *et al*. (2011) have shown that the positive effect of higher CO_2_ concentrations (on, e.g., N_2_ fixation) is primarily seen at lower light intensities, and Rijkenberg *et al*. (2011) stress that the stimulations expected may be counteracted by decreased supply of nutrients from deeper waters as a consequence of enhanced stratification.

## Conclusions and future outlooks

In 1968, Fay *et al*. (1968) proposed that the reducing conditions in the cyanobacterial cell type recognized as ‘heterocysts’ could be the site for N_2_ fixation. In 1973, Fleming & Haselkorn (1973) were the first to isolate nitrogenase from such heterocysts. Hence, heterocysts have during four decades acted as the ‘consensus model’ for successful *light-driven* N_2_ fixation in cyanobacteria. In 1991, Bergman and Carpenter were the first to show that nitrogenase is localized in subsets or short strings of cells (diazocyte) in *Trichodesmium*. These are now recognized as a prerequisite for the light-driven N_2_ fixation in *Trichodesmium*. This developmental mechanism is combined with mechanisms temporarily lowering oxygen evolution and orchestrating energy-competing processes in a multifaceted fashion. Hence, *Trichodesmium* is ‘second-to-none’ among potent daytime N_2_ fixers, as is in particular evidenced by its great global ecological impact. However, many questions still remain. These include identification of genes/proteins that underpin the development of the diazocytes and their regulation, including mechanisms involved in the protection of the oxygen-sensitive nitrogenase. Because freshwater species exist within the (former) genus *Katagnymene* (see e.g. Komárek & Anagnostidis, 2005), an intriguing question is whether these are capable of developing diazocytes. Sequencing additional genomes within this globally important genus will allow comparative genomic analyses and potentially shed light on, for example, its uniquely low DNA coding proportion and the significance of its apparently expanding genome. Besides genomic analyses, other ‘omics’ and ‘meta-omics’ approaches (transcriptomics, proteomics, and metabolomics) need to be introduced if we are to comprehend the unique N_2_-fixing physiology of *Trichodesmium* at all organization levels. ‘Meta-omics’ may provide more accurate information pertaining to the genetic diversity and the role of *Trichodesmium* and associated microorganisms than cultures, which often represents a minor part of the total species radiation. Such data may also reveal mutual and potentially life-sustaining interplays between *Trichodesmium* and the numerous associated microorganisms and open exciting research avenues into microbial evolution and marine microbial interphylum interactions, some potentially of a symbiotic nature. Focus should also be given to proteins that are highly up-regulated under N_2_-fixing conditions (Hewson *et al*., 2009; Sandh *et al*., 2011). Another area in need of exploration is the identification of adaptive ecological strategies, and possible niche differentiations, used by natural *Trichodesmium* populations. Finally, evolutionary aspects related to the uniquely different diazotrophic behavior of the genus *Trichodesmium* and to its placement in the evolution of affiliated cyanobacteria with unusual diazotrophic behavior are yet other compelling research areas to explore.

## Statement

Re-use of this article is permitted in accordance with the Terms and Conditions set out at http://wileyonlinelibrary.com/onlineopen#OnlineOpen_Terms

## Supporting Information

**Fig. S1.** The *Trichodesmium nif* region.

**Table S1.** Gene orthologs related to heterocyst differentiation.
